# Multi-scale measurement of stiffness in the developing ferret brain

**DOI:** 10.1038/s41598-023-47900-4

**Published:** 2023-11-23

**Authors:** Christopher Walter, Ramin Balouchzadeh, Kara E. Garcia, Christopher D. Kroenke, Amit Pathak, Philip V. Bayly

**Affiliations:** 1 Mechanical Engineering and Materials Science, Washington University, St. Louis, USA; 2 Radiology and Imaging Sciences, Indiana University School of Medicine, Evansville, IN USA; 3 Advanced Imaging Research Center and Oregon National Primate Research Center Division of Neuroscience, Oregon Health and Science University, Portland, OR USA

**Keywords:** Rheology, Atomic force microscopy, Development of the nervous system, Brain

## Abstract

Cortical folding is an important process during brain development, and aberrant folding is linked to disorders such as autism and schizophrenia. Changes in cell numbers, size, and morphology have been proposed to exert forces that control the folding process, but these changes may also influence the mechanical properties of developing brain tissue. Currently, the changes in tissue stiffness during brain folding are unknown. Here, we report stiffness in the developing ferret brain across multiple length scales, emphasizing changes in folding cortical tissue. Using rheometry to measure the bulk properties of brain tissue, we found that overall brain stiffness increases with age over the period of cortical folding. Using atomic force microscopy to target the cortical plate, we found that the occipital cortex increases in stiffness as well as stiffness heterogeneity over the course of development and folding. These findings can help to elucidate the mechanics of the cortical folding process by clarifying the concurrent evolution of tissue properties.

One of the most notable features of the human brain is the convoluted surface of the cerebral cortex. In healthy individuals, major, or primary, sulci (inward folds) and gyri (outward folds) tend to be consistently positioned and linked to known functional regions. By contrast, atypical folding can be observed in the secondary and tertiary folds of individuals affected by neurodevelopmental disorders, such as autism, schizophrenia, and Williams syndrome (WS)^1–5^. Cellular processes such as altered proliferative activity, cell differentiation, and/or establishment of synaptic connections^6–9^ have been implicated in normal and abnormal brain folding. However, it remains unclear how these processes translate to mechanical forces or properties that alter brain morphology.

Modeling and computational studies have offered important insights into the physical process of brain folding, finding that cortical expansion and the resulting mechanical instability is sufficient to explain many observed features of cortical folding. However, accurate simulations require realistic mechanical properties such as brain viscoelasticity, differential stiffness between the cortex and white matter, and anisotropy within white matter^5,10–16^. Previous studies have measured mechanical properties of fully-developed, adult brain tissue in humans and various other species^17–33^. Additionally, studies have suggested that brain tissue stiffness changes in concert with certain developmental processes, most notably with the maturation of myelinated axonal tracts^4,34–37^. Rheological techniques, such as shear rheometry, have been employed previously to measure the mechanical properties of brain tissue, such as shear modulus, at the macroscopic level^26,31,38^. By contrast, atomic force microscopy (AFM) has been employed to probe local mechanical properties of a variety of materials, including brain^39–43^ and other biological tissues^41,44,45^, with high spatial resolution.

Despite these advances, the mechanical properties of brain tissue over the period when gyri and sulci are forming have not yet been explored^46^. In this study, we report mechanical properties of brain tissue over the developmental period of brain folding in the ferret (*Mustela furo*), a gyrencephalic rodent in which brain folding occurs postnatally. Considering brain specimens derived from animals ranging in age from 8 to 39 postnatal days (P8-P39), as well as tissue from adult animals, we compare bulk measurement of biomechanical properties from rheometry with microscale measurements of mechanical properties of the developing occipital cortex via AFM. This combination of techniques provides an in-depth look at the dynamic changes of brain material properties to develop a greater understanding of their role in cortical folding.

## Results

### Specimen selection for mechanical characterization of developing brain tissue

Macroscopic and microscale measures of brain tissue were obtained from a total of 52 brain specimens, representing 46 ferret kits (25 male, 21 female) at various stages of brain folding, as well as 6 adult jills (all female). As described in Table 1, this included 7–8 kits across each of the following age ranges: P8-10, P13-16, P20-22, P26-29, P31-33, P38-39, as well as 1 kit aged P45. Approximate balance between male and female specimens and litter was maintained at each developmental age range. Following the procedure described in Fig. 1, 18 specimens were included in both AFM and bulk rheometry studies, and 3 specimens were included in both AFM and bulk water content studies.

**Table 1:** Animals used for AFM, rheometry and water content measurements.

#	Age (Days, P-)	Sex	Weight (g)	Rheometry (Used, # Slices)	AFM (Used, # Slices, # Force Maps)	Water content (Used, # Samples)
F1	8	M	24.9	X, 1	X, 1, 1	
F2	8	F	39.3	X, 1		
F3	8	M	28.6	X, 1		
F4	8	F	28.7	X, 2		
F5	8	M	31			X, 1
F6	8	F	41			X, 1
F7	10	F	36	X, 1	X, 1, 2	
F8	13	F	40	X, 2	X, 1, 2	
F9	13	F	68			X, 1
F10	14	F	53	X, 2		
F11	14	M	70	X, 1		
F12	14	M	70	X, 2		
F13	14	M	66.1		X, 1, 2	
F14	14	M	91			X, 1
F15	16	F	79	X, 4		
F16	20	M	111	X, 2	X, 1, 2	
F17	20	M	120	X, 2	X, 1, 2	
F18	21	F	107	X, 2	X, 1, 2	
F19	21	M	130	X, 2	X, 1, 1	
F20	21	F	95.5	X, 2	X, 1, 1	
F21	21	M	61			X, 1
F22	22	F	68		X, 1, 2	X, 1
F23	26	M	183	X, 3	X, 1, 1	
F24	26	M	126			X, 1
F25	27	M	181	X, 2	X, 1, 1	
F26	27	F	160	X, 3		
F27	27	M	204	X, 2	X, 1, 1	
F28	27	F	146.6		X, 1, 2	
F29	27	M	215			X, 1
F30	29	M	220	X, 4		
F31	31	M	201	X, 4		
F32	31	F	157	X, 4		
F33	31	F	220	X, 3	X, 1, 1	
F34	32	M	270	X, 3	X, 1, 1	
F35	32	M	195			X, 1
F36	33	M	243	X, 3		
F37	33	F	232		X, 1, 1	
F38	33	M	292		X, 1, 1	X, 1
F39	38	M	175	X, 2	X, 1, 1	
F40	38	F	180	X, 4	X, 1, 1	
F41	38	M	334	X, 4		
F42	39	F	300	X, 2	X, 1, 1	
F43	39	F	307	X, 4		
F44	39	M	400		X, 1, 1	
F45	39	F	255		X, 1, 1	X, 1
F46	45	F	380			X, 2
F47	Adult	F	789	X, 3	X, 1, 1	
F48	Adult	F	800	X, 4		
F49	Adult	F	819	X, 4		
F50	Adult	F	756	X, 3	X, 1, 1	
F51	Adult	F	818	X, 4		
F52	Adult	F	826			X, 1

**Figure 1:**
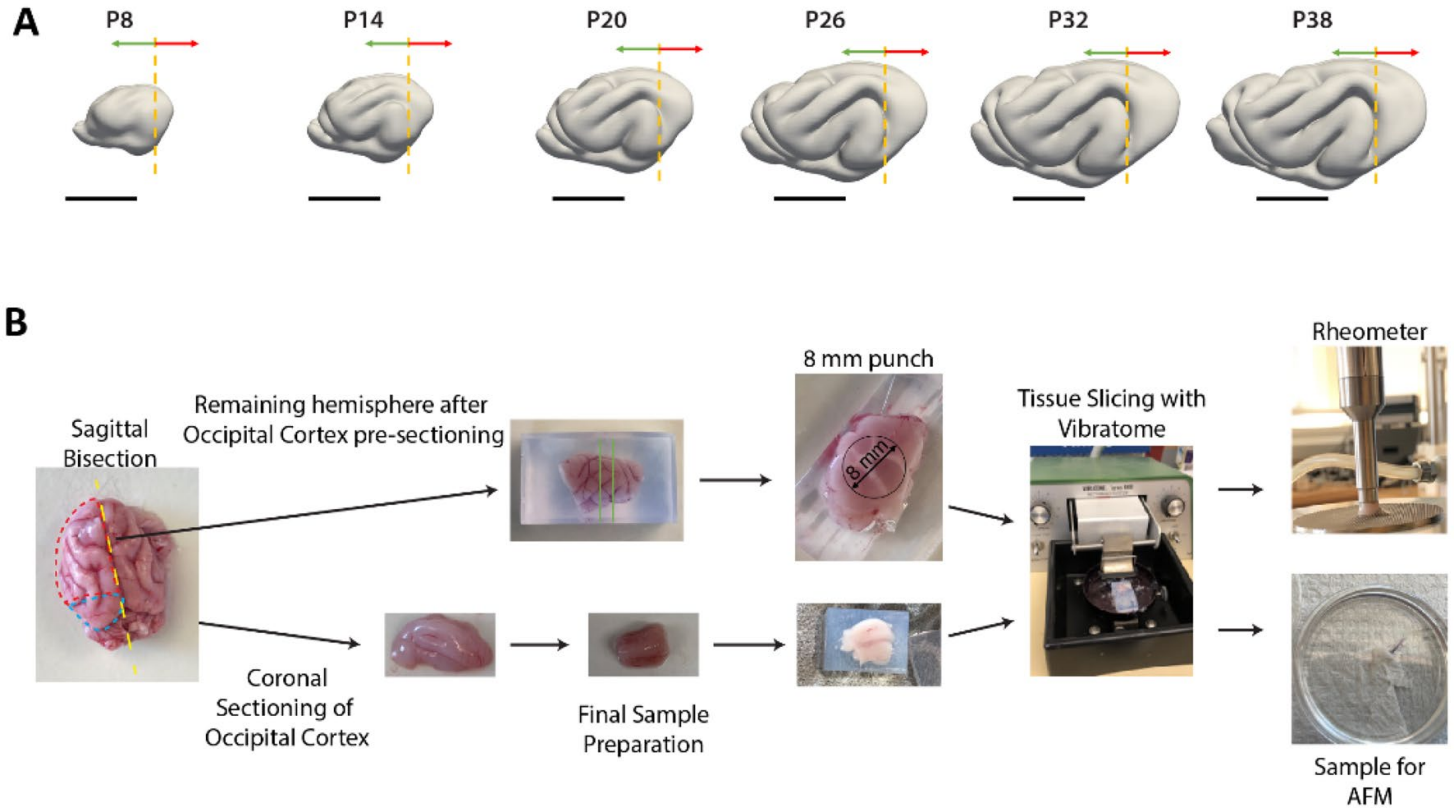
Representative rheometry and AFM tissue slice preparation diagram. (**A**) Representative cortical surface models of ferret brain folding at developmental time points derived through magnetic resonance imaging. Dashed line indicates how brain was divided for testing with rheometry (middle and anterior portions, denoted with green arrow) versus AFM (posterior portion, denoted with red arrow). Scale bar = 10 mm. (**B**) Representative workflow depicting preparation of tissue samples for rheometry and AFM. Full details for sample preparation are located within the materials and methods section.

### Rheometry reveals increased brain tissue stiffness with age

**Figure 2:**
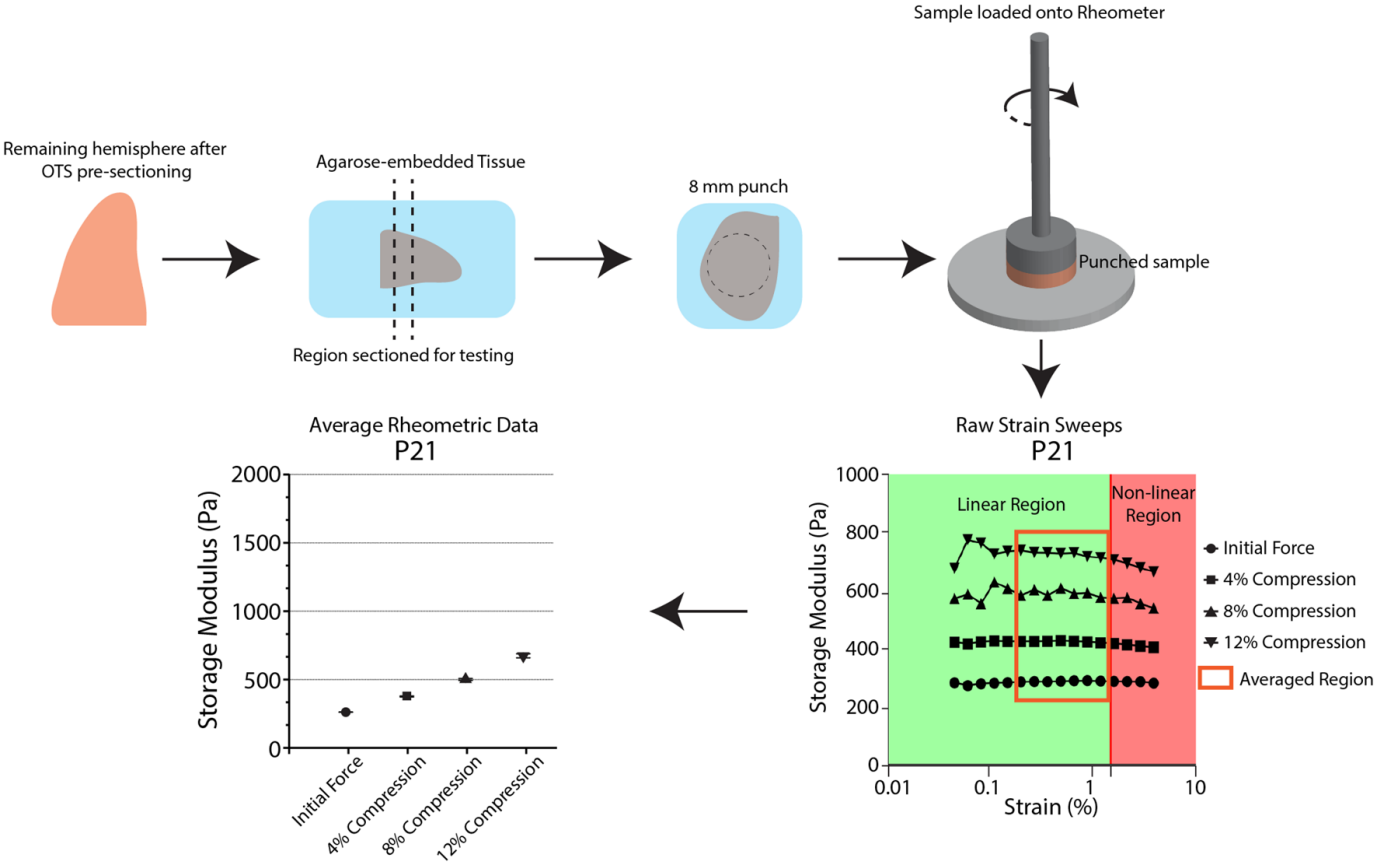
Determining bulk brain stiffness via rheometry. Example workflow of rheometry experiment from sample preparation to rheometry, with example raw strain sweeps indicating the range of strains considered for this portion of the study. This section is selected to avoid non-linear effects from insufficient or excessive strains. These data are then averaged to generate the individual reported moduli per sample at the given imposed compression. Further details can be found in Supplementary Figure S1 and the Materials and Methods.

As illustrated in Fig. 1A, following hemisection of the brain, hemispheres were blocked with a single coronal cut to separate the occipital lobe from the rest of the brain. Coronal slices of ~ 3 mm thickness (Fig. 2) were prepared using a vibratome from the bulk brain tissue rostral to the initial coronal cut, and slices were trimmed to be compatible with the rheometer probe shape using an 8 mm diameter tissue punch (Fig. 1B). Rheometry was performed on 1 to 4 slices (average of 3) per animal, with an average slice thickness ($\pm$ std. dev.) of 2.80 $\pm$ 0.49 mm.

**Figure 3:**
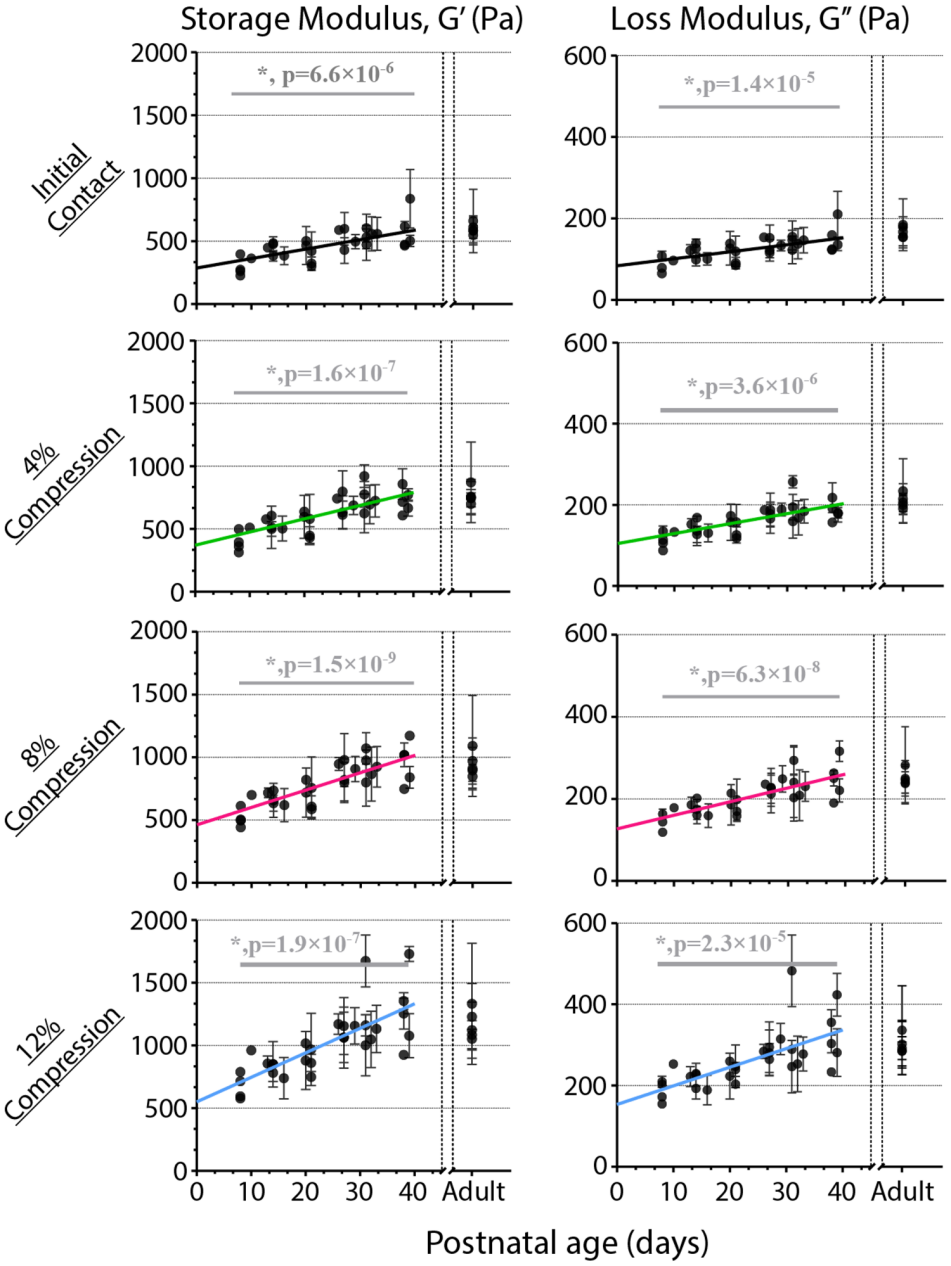
Elastic and viscous components of bulk brain stiffness increase with compression and age. Storage (${G}^{\prime}$, left) and Loss (${G}^{{\prime}{\prime}}$, right) moduli for 4 different initial compressive values for rheometry. Data points represent individual sample from a single hemisphere of a subject. Error bars = SD. Lines represent fit based on linear mixed-effects model. N ≥ 3.

Rheometry was performed by applying torsional shear to the tissue after establishing minimal contact and at various levels of subsequent compression. Data were analyzed from strain sweeps (0.1–1.0% strain, Fig. 2, Supplementary Fig. S1) at 0.1 Hz at four different levels of compression: initial contact, 4%, 8%, and 12% (Fig. 3, Supplementary Fig. S2A). In general, both the estimated storage modulus (${G}^{\prime}$*,* the elastic component of the complex shear modulus) and loss modulus (${G}^{{\prime}{\prime}}$*,* the viscous component) increased with increasing compression. At each compression level we observed increases in both ${G}^{\prime}$ and ${G}^{\prime\prime}$ throughout brain development (Fig. 3, Supplementary Fig. S2A). The rate of increase in the storage modulus, ${G}^{\prime}$, calculated through a linear mixed-effects model, was 7.53 Pa/day for samples in initial contact (minimal compression), and 10.49, 13.85, and 19.45 Pa/day for 4%, 8%, and 12% compression, respectively. The increases of ${G}^{\prime}$ with age were found to be statistically significant for all compression levels. The loss modulus, ${G}^{{\prime}{\prime}}$ also increased in an age-dependent manner during development, at a proportionally slower rate compared to ${G}^{\prime}$. The rates of increase in ${G}^{\prime\prime}$ for the initial contact, 4% compression, 8% compression, and 12% compression tests were 1.96, 2.62, 3.37, and 4.64 Pa/day, respectively (all statistically significant). Interestingly, the dependence of resistance to shear (${G}^{\prime}$ and ${G}^{{\prime}{\prime}}$) on compression appeared to increase with age. At the youngest age tested (P8), ${G}^{\prime}$ and ${G}^{{\prime}{\prime}}$ increased in parallel with compression level, with differences between initial contact and 12% compression ($\Delta {G}^{\prime}$ and $\Delta {G}^{{\prime}{\prime}}$) of approximately 0.35 kPa and 0.10 kPa, respectively (Supplementary Fig. S2B). This disparity between the moduli at the greatest and least compression increased with age, up to nearly 0.71 kPa and 0.19 kPa for $\Delta {G}^{\prime}$ and $\Delta {G}^{{\prime}{\prime}}$ respectively, at P39 (Supplementary Fig. S2B).

### Brain stiffness in the folding occipital cortex increases with age

**Figure 4:**
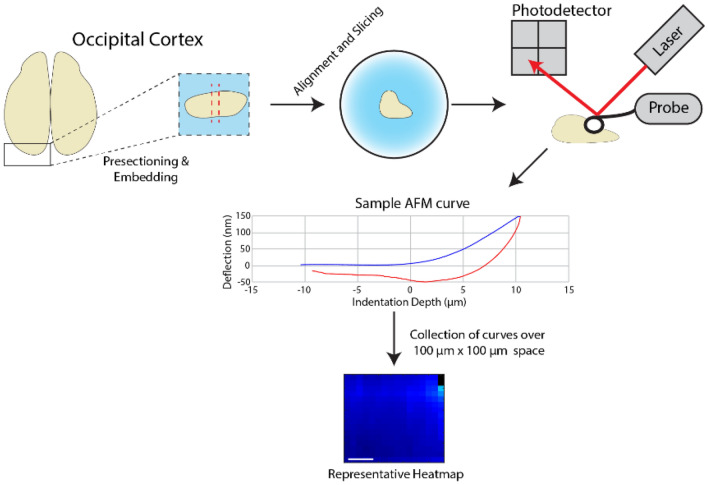
Measurement of microscale OC stiffness via AFM. Representative workflow diagram for sectioning of ferret brain occipital cortex (OC) and testing via AFM, resulting in a collection of force curves and modulus heatmaps.

In addition to measuring changes to overall brain stiffness throughout development, we sought to investigate the changes to microscale stiffness in the developing cerebral cortex. The occipital cortex (OC) region was chosen for AFM measurements, in part because the occipital temporal sulcus (OTS) forms within this region over the age range investigated. Occipital lobes were dissected from the ferret brain as shown in Fig. 4, embedded in agarose, sliced at a 300 μm thickness on a vibratome, and aligned on an AFM (Bruker, Billerica, USA) (Figs. 1B, 4) Indentations were taken in the immediate vicinity of the OTS when it emerged (ages ≥ P26), or from the region expected to develop into the OTS in samples from younger animals (Fig. 4). In total, AFM data was obtained from 23 kits (12 male) and 2 adult females, from 1 slice in the approximate same location per sample (see Materials and Methods), and 1 to 2 force maps per slice.

**Figure 5:**
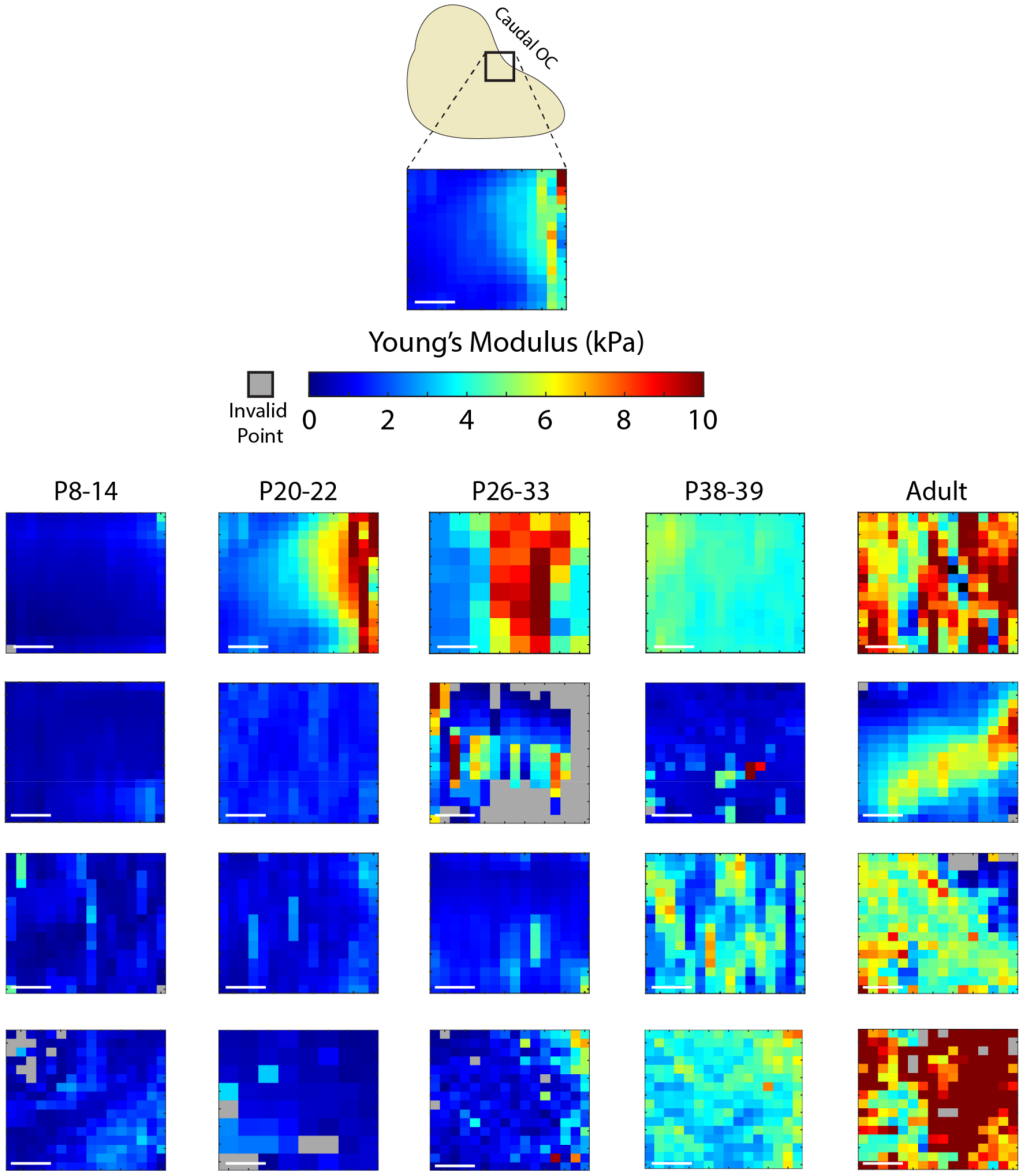
Heterogeneity in OC stiffness increases with postnatal ferret age. Representative heatmaps of Young’s modulus obtained via AFM across tissue slices. All heatmaps are the same overall size (100 µm × 100 µm) with the same orientation and tissue location, indicated in the top diagram. Ages are indicated above heatmaps. Individual blocks indicate Young’s modulus tabulated from an indentation at that point on the tissue. Scale bars = 25 µm.

**Figure 6:**
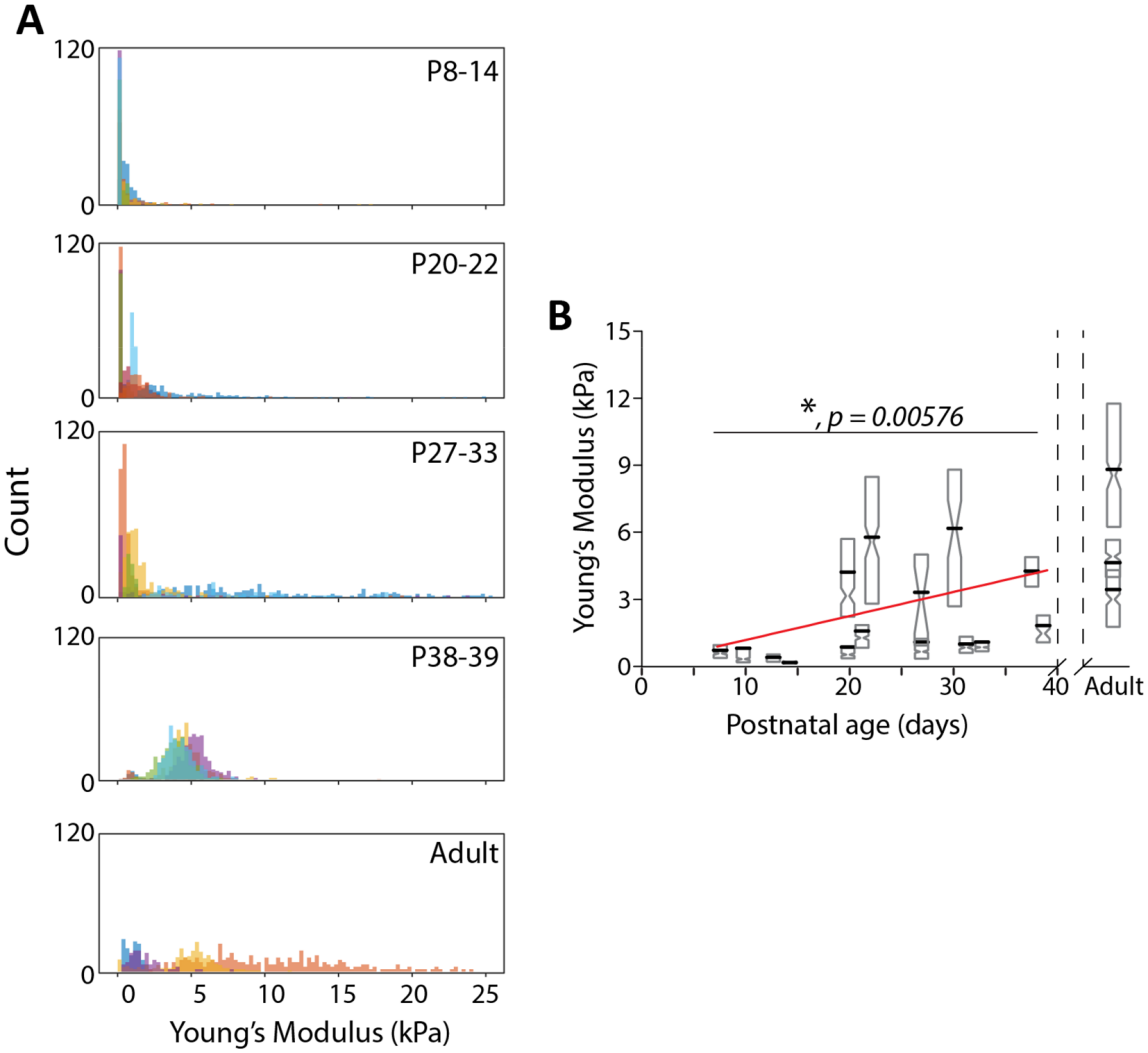
OC stiffness increases and distribution of OC stiffness shifts with increasing postnatal ferret age (**A**) Individual histograms of Young’s Moduli ($E$) of ferret brains at increasing post-natal age ranges. Bin widths are 250 Pa. Different colors represent different samples. (**B**) $E$ of ferret brains at increasing postnatal age. Grey diamond boxes represent complete set of accepted $E$ values per sample. Upper and lower bounds of boxes represent standard deviation with the pinch point at median value. Black bars indicate mean $E$. Red line indicates linear fit generated by linear mixed-effects model from all values. * indicates statistically significant increase in $E$ with increase in postnatal age. P value listed on plot.

As with bulk brain tissue, AFM-determined tissue stiffness in the OC increased with age (Figs. 5,6). At early stages of development, the variance in stiffness was relatively low, and very few regions exhibited Young’s modulus,$E$, above 1 kPa (Fig. 5). At P20, while still soft, stiffness was noticeably higher while handling the tissue, and Young’s modulus in these tissue samples was observed to be approximately 2.5 kPa. This increase in stiffness held at ages beyond P20, with average $E$ reaching values of 6 kPa in some animals (Fig. 6). Brains from ferrets at P26 and beyond exhibited extensive signs of brain folding, including OTS development visible without the aid of any visualization equipment (Fig. 1B). However, not all animals at ages above P20 showed large $E$ values, with animals at P27, P32, and P33 having average $E$ of ~ 1.5 kPa (Fig. 6). This variation in $E$ could be due to variation in brain development among individual animals, or inevitable, small methodological variations (slicing, storage). Despite this, we noted fewer regions of stiffness below 1 kPa and greater variance in OC stiffness in ferrets age P20 or older (Figs. 5, 6). These trends held in adult ferrets, in which brains were generally stiffer than in the juveniles. Linear mixed-effects analyses demonstrated that the observed increase was statistically significant ($p=0.006$). Furthermore, using this model, we were able to estimate that OC stiffness increased by approximately 0.1 kPa per day.

### Age-dependent increase in brain stiffness is correlated across length scales

To see how well bulk-scale mechanical properties correlated with the microscale, we compared data obtained via AFM and rheometry using samples from the same animal. This resulted in comparison of 16 kits (9 male) + 2 adult females, with an average of 2.5 rheometry slices and 1.2 AFM samples per ferret. To compare the two methods, we first calculated an effective Young’s modulus (${E}_{eff}$) from the storage and loss moduli measured via rheometry. We began by calculating the magnitude of the complex shear modulus (*G*) from the magnitudes of the storage and loss moduli (Eq. 1).

1 $$G= \sqrt{{G^{\prime}}^{2}+{{G}^{{\prime}{\prime}}}^{2}}$$

We then estimate the ${E}_{eff}$ from *G* and the Poisson’s ratio (ν) of the tissue (Eq. 2).

2 $${E}_{eff}= 2G(1+\upnu )$$

**Figure 7:**
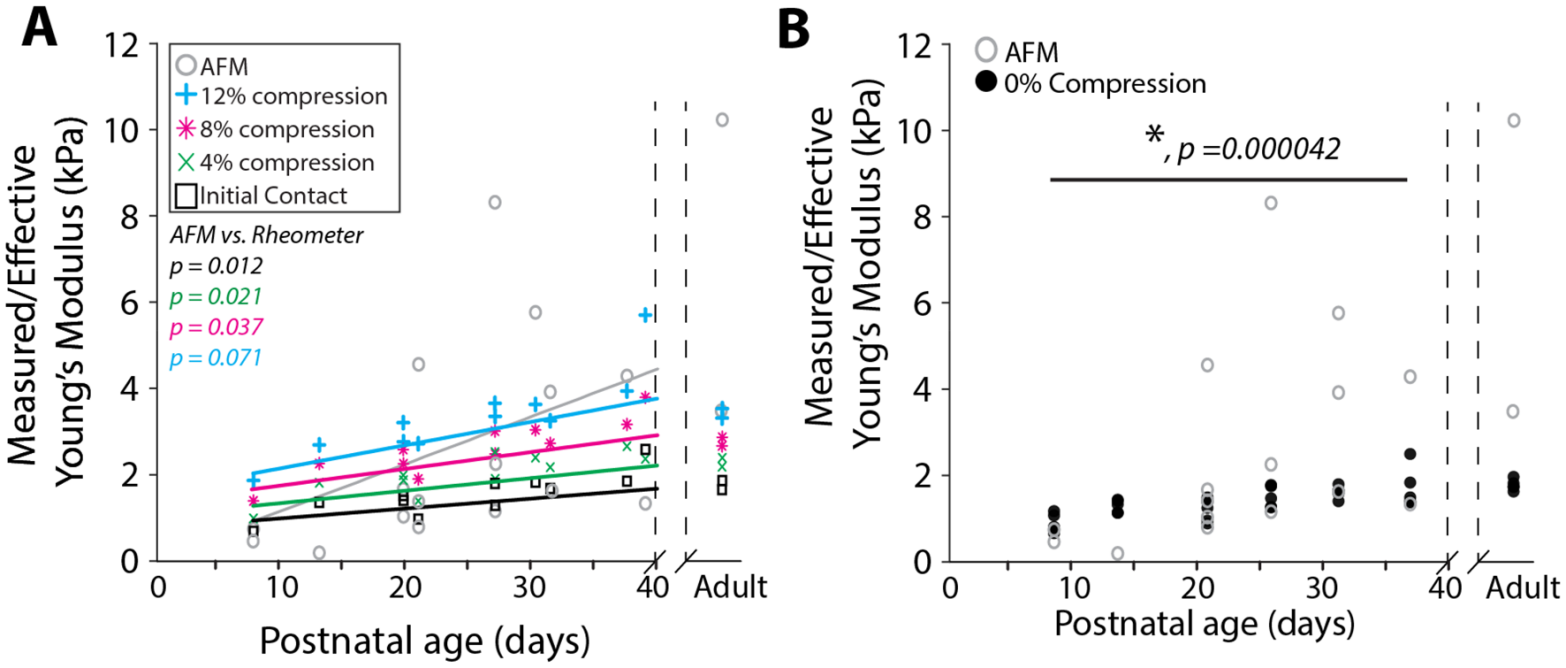
Comparison of rheometric and AFM measures of developing brain stiffness reveal disparity between bulk and microscale tissue development. (**A**) Comparison of mean Young’s moduli measured (AFM) or estimated from shear modulus (rheometry) in specific animals used for both AFM and rheometry experiments. Each data point represents the average value for one animal. Fitted lines are generated from a linear mixed-effects model. P-values are indicated on the left-hand side of the plot, comparing AFM to the corresponding rheometric test. (**B**) Estimates of shear modulus at 0% compression estimated by linear regression model from measured moduli at 4%, 8%, and 12% compression, with AFM. Each data point represents the average value for one animal.

**Figure 8:**
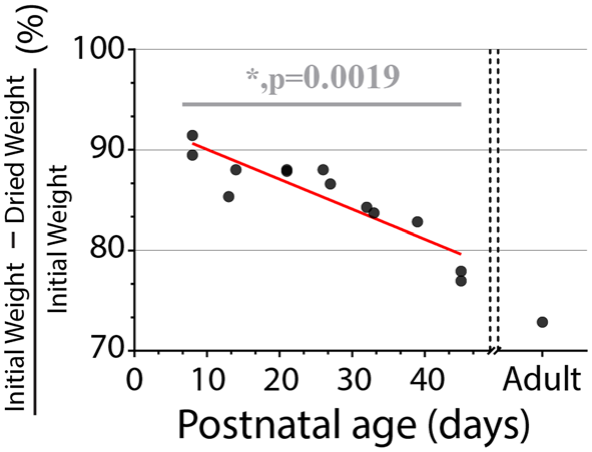
Water content of ferret brain tissue decreases with age. Water content of ferret brain hemispheres measured from the difference in weight before and after drying. The fitted line is obtained from a linear mixed-effects model.

For biological tissue, and with small deformations, a Poisson’s ratio of 0.5, that of an incompressible material, is commonly assumed^47,48^ to simplify the equation to ${E}_{eff}\approx 3G$. The global estimate of Young’s modulus (${E}_{eff}$) from rheometry at the four compression levels, as well as the measured *E* from AFM, are plotted together in Fig. 8. While estimates of $E$ at both scales increase with age, the increase in stiffness with age is more subtle in the tissue-scale (rheometry) measurements. Results from rheometry are closer to those from AFM at younger ages. All tests combined show a strong correlation of tissue stiffness with age ($p=0.03$), and a weak correlation of local estimates of Young’s modulus obtained by AFM to global estimates obtained through rheometry ($p=0.05$) (Fig. 7A). Additionally, rheometry on adult tissue led to estimates of Young’s modulus lower than those measured via AFM (Fig. 7A).

For a more consistent comparison of AFM and rheometry data, we used data from higher levels of compression to estimate an effective modulus at 0% compression. AFM deformations are limited to a maximum of 250 nm, therefore minimal compression would be induced into the tissue during those experiments. We found that the estimated modulus at 0% compression was the most highly correlated with AFM measurements ($p=0.00004$), and the correlation between AFM and rheometry decreases with the level of imposed compression during rheometry (Fig. 7A,B).

Finally, we compared the rates of increase in moduli across both testing methods. For rheometry, ${E}_{eff}$ increased by a minimum of ~ 25 Pa/day, at initial contact, to a maximum of ~ 60 Pa/day, at 12% compression (Supplementary Fig. S3). By comparison, AFM estimates of Young’s modulus increased by approximately 100 Pa/day.

Together, these data show that estimates of both global brain stiffness from rheometry and local cortical stiffness from AFM show similar overall trends with age; however they describe different features of brain tissue, both of which are likely important for understanding brain development.

### Water content of brain decreases with development/age/brain folding

Finally, we speculated that the observed increase in ferret brain tissue stiffness with development may be related to changes in the water content of the brain over the first postnatal weeks. Water content has been proposed to play a crucial role in determining the mechanical properties of biological tissues, including the brain, as it is a major component of cells and extracellular matrix^49–51^. To investigate the change in water content of the brain tissue throughout folding, bulk samples (average: 1.1 samples/animal) from 12 kits (7 male) and 1 adult female were weighed before and after 3 days of drying at 39 °C. We found a clear decrease in water content at a rate of 0.3% /day (w/w) from the postnatal ages of 8–45 days (Fig. 8). This decline in water content with age coincides with increases in both the elastic and viscous moduli of the tissue and presumably is intrinsic to brain development.

## Discussion

In this study, we have shown that the stiffness of the ferret brain increases throughout neonatal development, both globally and locally within the cortical plate of the developing OC. Through rheometry, we find that both the global elastic and viscous resistance of brain tissue to shear increases over the first 40 postnatal days, reaching adult levels (0.5–1.4 kPa, depending on imposed compression) consistent with past measures of adult cortical brain tissue (1–2 kPa)^26,31,52^. Furthermore, we find that resistance to shear deformation increases with imposed compression. These stiffness changes are coincident with documented increased production of ECM proteins and cell differentiation and proliferation in the brain^53,54^. Additionally, we present novel findings of increasing microscale stiffness of the developing occipital cortex during the cortical folding process, measured through AFM. In analogous studies, magnetic resonance elastography has been utilized to characterize the viscoelasticity of whole ferret brains during the cortical folding process, however direct comparison with elastic stiffness measured through AFM is difficult^32,55,56^. The stiffness of embryonic mice^57,58^ and songbird brains^58^ have been reported prior to the cortical folding process, with Young’s moduli on the order of hundreds of Pa, similar to the youngest ages tested in this study. Microscale stiffness of adult mouse brains^40,52,59^ showed greater variation, with values ranging from hundreds of Pa to tens of kPa, in further agreement with our findings.

Despite a general concordance with an increase in both the Young’s modulus (measured by AFM) and the shear modulus (measured by rheometry), the two methods of measuring tissue properties are sensitive to different aspects of the tissue development. The particular benefit to AFM is its ability to measure mechanical properties of smaller target regions, which enables characterization of specific brain regions, such as areas of the cortical plate. As illustrated in the heatmaps of Fig. 5, AFM also has the potential to reveal heterogeneities within the cortex at high spatial resolution. Future work should examine whether specific cellular components (e.g., individual axons or dendrites) or tissue level features (e.g., blood vessels) underlie within-sample heterogeneities observed in the current study.

Together, differences between modulus estimates from the two methods highlight the different behavior of the tissue probed by global rheometry and local AFM measurements, arguing for the use of both to robustly characterize soft tissue development. These estimates of shear and elastic moduli in the developing brain are critical to building accurate computational models of cortical folding. For example, current models have suggested cortical expansion as a driving force for cortical folding, inducing mechanical instability (buckling) in the form of wrinkling or creasing. However, in models considering purely elastic or hyperelastic properties, the ratio of cortical to subcortical stiffness can dramatically impact the shape^60,61^ and wavelength of folds^62,63^. Similarly, heterogeneities in cortical stiffness have been proposed to impact the locations of folds^4^. In this study, we find that Young’s modulus within the cortex, based on linear regression fit of AFM data, ranges from 1 to 2.5 times the estimated Young’s modulus of bulk tissue, which includes both cortex and subcortical tissues. Therefore, it is unlikely that large, order-of-magnitude differences exist in the elastic properties of different brain tissues. (Approximating bulk samples to contain half cortical tissue, cortical stiffness ranges 1 to 4 times the stiffness of subcortical layers.) Future models may benefit from these bounding parameters. However, it is worth noting that the behavior of both cortical and subcortical tissue is likely more complex, including anisotropic and growth behaviors not considered here^6,25,31,36,64,65^.

In addition to quantification of global and microscale tissue stiffness via rheometry and AFM, respectively, we showed that the water content of brain tissue decreases significantly during development, potentially from the tradeoff of interstitial fluid for developed ECM structure and cell content. Water content plays a crucial role in influencing the mechanical properties of biological tissues, including the brain, as it is a major component of cells and extracellular matrix. Water content affects tissue elasticity and viscosity. For example, a study by Gefen et al.^66^ found that increased water content in brain tissue resulted in reduced stiffness, leading to higher susceptibility to mechanical deformations^66^. Similarly, another study by Elkin et al.^67^ demonstrated that changes in water content within the brain altered the viscoelastic properties, affecting the tissue's ability to dissipate mechanical energy during loading^67^. These findings highlight that both the tissue itself and its ability to retain water contribute significantly to overall brain stiffness and folding, emphasizing the need for better understanding of its responsibility for brain health and function. Future experiments could further elucidate the observed relationship we have reported here by varying osmotic conditions for sample testing, however that is beyond the scope of this study.

In this study, we report global (from rheometry) and local (from AFM) changes in stiffness of brain tissue during development and folding in a gyrencephalic animal. The requirement to test fresh tissue poses a challenge to the use of AFM. Fresh brain tissue is very soft and difficult to slice, especially for the softer early postnatal ages. Consequent tissue damage and thickness variations may underlie some of the variability in AFM, and further motivate the complementary rheometer measurements. Despite this challenge, the combined methods allow us to probe micro-scale and global changes in tissue stiffness in developing brain tissue. This approach may be used to study changes in mechanical properties across cortical regions and different brain tissues throughout development.

## Materials and methods

### Ferret handling and brain extractions

All animal procedures were conducted in accordance with the relevant guidelines and regulations of the Public Health Service Policy on Human Care and Use of Laboratory Animals and American Veterinary Medical Association Guidelines for the Euthanasia of Animals. All animal procedures were approved by the Washington University Institutional Animal Care and Use Committee and all experimental methods were exactly followed in accordance with the approved guidelines and regulations. This study was conducted in accordance with the ARRIVE guidelines. Data were acquired from a total of 46 ferret kits (25 male, 21 female) and 6 adult jills (all female). Animals were delivered to Washington University from Marshall Bioresources on day P5. Each ferret used in this study was euthanized by sodium pentobarbital overdose. Extracted brains were then submerged into artificial Cerebrospinal Fluid (aCSF), consisting of: 1 × Hank’s Balanced Salt Solution with 0.25 mM HEPES, 3 mM D-glucose, 0.2% (v/v) Phenol Red, and the following added salts: 1 mM CaCl_2_, 1 mM MgSO_4_, and 4 mM NaHCO_3_, immediately after extraction and kept in a 4 °C temperature environment until ready for testing preparation. A full breakdown of the animal usage in this study is provided in Table 1.

### Brain tissue preparation and slicing

The brain tissue processing workflow is shown in Fig. 1. Brains were initially bisected into two hemispheres along the sagittal plane. The most caudal sections where the OC is located were then removed via razor blade through a coronal cut. Tissue rostral to the cut was utilized for rheometry, and tissue caudal to the cut was further sectioned for AFM. For preparing samples for rheometry, sections of 3 mm thickness were generated using a vibratome (Fig. 1), and these were subsequently punched with an 8 mm diameter circular punch and transferred to the rheometer for testing. Because samples are quite large relative to brain size, they typically contain both gray matter (cortical and subcortical) and developing white matter. For AFM, the OC region was embedded in 0.75% low melting point agarose (Thermo Fisher) and allowed to set for 20 min at 4 °C. The excess agarose gel was then removed and the brain section embedded in agarose is positioned on a vibratome such that the blade cut through the OC in the medial to lateral direction in the horizontal plane. The vibratome blade was covered in aCSF prior to slicing to both aid in generating slices and transferring the tissue from the vibratome to the sample dish, as well as provide a surface layer of aCSF to prevent excessive tissue drying while samples were allowed to attach to the dish surfaces. Preparatory slices were then taken until the topmost layer was removed, followed by two-1 mm slices through the most medial portions of the tissue section to ensure similar location of testing within the OC of the tissue at each age. A 300-µm slice was then made and transferred to a CellTak-treated (Thermo Fisher) glass bottom petri dish (WillCo) and left for 5 min to bond to the surface. This step is necessary to allow firm attachment to the dish surface for AFM measurements; it did not appear to cause tissue drying or loss of integrity. Following attachment to the petri dish surface, the excess agarose around the tissue section was then removed and the sample was covered in aCSF and immediately transferred to the AFM for stiffness measurements (Fig. 1). In total, samples are typically dissected, sliced and loaded for testing within 90 min, in order to maintain sample integrity and to limit the potential changes in brain tissue mechanical properties post mortem.

### Bulk brain stiffness measurement via rheometry

Tissue samples were loaded onto the rheometer (Discovery Hybrid HR-20, TA Instruments, New Castle, DE) equipped with a cross-hatched baseplate and an 8 mm cross-hatched loading surface (“geometry”). Samples were placed in the center of the plate and kept hydrated during testing by adding droplets of aCSF around the sample. (Fig. 1). Temperature was held at 23 °C with the rheometer Peltier plate for the duration of the tests.

Rheometry experiments were performed by applying torsional shear to the sample while it was compressed between the surfaces of the loading geometry and the baseplate. First, initial contact with the tissue sample was established by reducing the gap between geometry and baseplate until a normal force of 0.01N was established. Torsional shear testing was performed at this initial gap, and again each time after the gap was reduced to achieve a compressive pre-strain of 4%, 8%, and 12%, respectively. At each level of pre-compression, a frequency sweep was performed from 0.05 to 0.5 Hz at 1% nominal shear strain, and strain sweep was performed from 0.1 to 10% shear strain at a frequency of 0.1 Hz. Raw phase was monitored to confirm that inertial effects were negligible relative to viscoelastic torques under these conditions. Data for analysis were selected from the strain sweep over the range in which the sample exhibited linear viscoelastic behavior (Fig. 2, Supplementary Fig. S1).

### Microscale OC stiffness measurement via atomic force microscopy

After tissue slice preparation, slices were moved to a Bruker Nanoscope Resolve atomic force microscope (Bruker, Billerica, USA). The stage and sample were kept at 37 °C throughout the experiment. Custom AFM probes were used with a silicon nitride cantilever (0.01 N/m reported stiffness) and a 4.5 µm polystyrene bead attached as a tip (Novascan Technologies, Inc., Boone, IA, USA). Tips were initially allowed to equilibrate in aCSF, which served as the testing buffer, for 10 min followed by calibration to determine the spring constant of the cantilever. The sample was located using the attached microscope (Carl Zeiss Microscopy, Germany) and force volumes were taken in 100 µm × 100 µm sections around this area. The maximum tissue indentation set point was set to 250 nm for all samples. Cantilevers were given a maximum range of 12 µm to approach the tissue surface, make contact with the surface, and reach maximum tissue indentation (Fig. 4). Cantilever approach speed was set to 1 µm/s to avoid skewing stiffness data due to viscous effects from testing samples in fluid. A modified Hertz model, implemented in Nanoscope Analysis Software (Bruker, Billerica, USA), was used to analyze the force curves and export stiffness data. A Poisson’s ratio of 0.5 was assumed in order to calculate elastic modulus from the Hertz model. A strict protocol was followed to select quality scans of the tissue. Typical force curve selection was performed, only analyzing and including data generated from force curves that had the following characteristics: (1) a flat approach curve, (2) an extension curve that displayed a steady increase in deflection with indentation, and (3) a return to 0 value upon full retraction, with expected sticking to the tissue (See Fig. 4 for example of a good force curve). Force volumes were then only selected if a region of at least 5 × 5 selected indentations were obtained. Multiple force volumes were taken per sample only when the OTS or edge of the tissue was unclear, typically in younger samples. Sample testing was concluded within 1.5 h from sample preparation (average total time post mortem: 2 h 45 min) to limit potential changes in brain tissue mechanical properties post mortem. As a result of these strict parameters, calculated Young’s Moduli did not fall outside of the range of 0.1–25 kPa, a reasonable range given the reported stiffness of the custom probes used. This also resulted in many samples not being considered for analysis and reported in this study. A full table detailing the ferrets used in this study and the measurement techniques recorded in this study is available in Table 1.

### Water content

Brain water content was measured in 13 ferrets spanning ages P8-P42 and adult. After brain extraction, one hemisphere of the brain, without cerebellum and olfactory bulb, was weighed $[{\left({\text{weight}}\right)}_{i}]$. Each sample was dried at 39 ± 1 °C for 3 days and weighed again ${[\left({\text{weight}}\right)}_{f}]$. The brain water content was calculated as follows:

$$\text{Water} \, \text{content} \left(\%\right)=\frac{{\left({\text{weight}}\right)}_{i}-{\left({\text{weight}}\right)}_{f}}{{\left({\text{weight}}\right)}_{i}}\times 100$$

### Statistical analysis

To account for within-animal correlations between measurements in the analysis of effects between individuals over the course of development, statistical analysis reported in this study utilized a mixed linear effects modeling strategy, implemented with the “lmefit” function in MATLAB. For rheometry, data from multiple slices were grouped by individual, and for AFM, individual scans were grouped by individual. Inter-individual differences were treated as a random effect, and age-dependent differences were treated as a fixed effect. The latter was displayed as linear trend lines in Figs. 3 and 6. In the calculations of statistical significance of age in Young’s modulus, the data from adult tissues were not included due to the unknown age of the adults, as the expected nonlinear dependence of stiffness on age subsequent to the developmental age range studied here (e.g., transition from increasing with age to no dependence on age).

### Supplementary Information

Supplementary Information.

## Data Availability

All datasets generated during and/or analyzed in this study is available upon reasonable request from the corresponding authors.
