# Supplementary material for: Multi-scale measurement of stiffness in the developing ferret brain
Source: Sci Rep. 2023 Nov 23;13:20583. doi: 10.1038/s41598-023-47900-4 (PMC10667369; doi:10.1038/s41598-023-47900-4)
Supplement: Supplementary file 1 — Supplementary Information. [file 41598_2023_47900_MOESM1_ESM.pdf]

Supplementary Figures

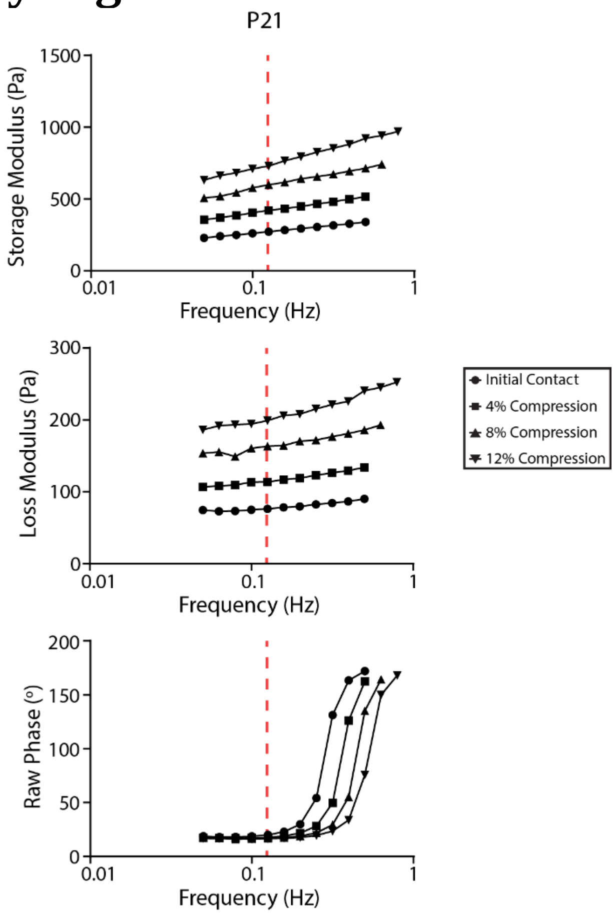

Supplementary Figure S1. Representative frequency sweeps establishing common test frequency for Rheology. The visual representation illustrates the selection of the frequency for rheometric strain sweeps. The red dashed line highlights the chosen frequency, which was selected to remain within the quasi-static region for measuring both the Storage Modulus and Loss Modulus. This choice was made in conjunction with a low Raw Phase value. Increases in the Raw Phase indicate a higher correction applied by the rheometer to the measured values, potentially complicating data analysis and interpretation.

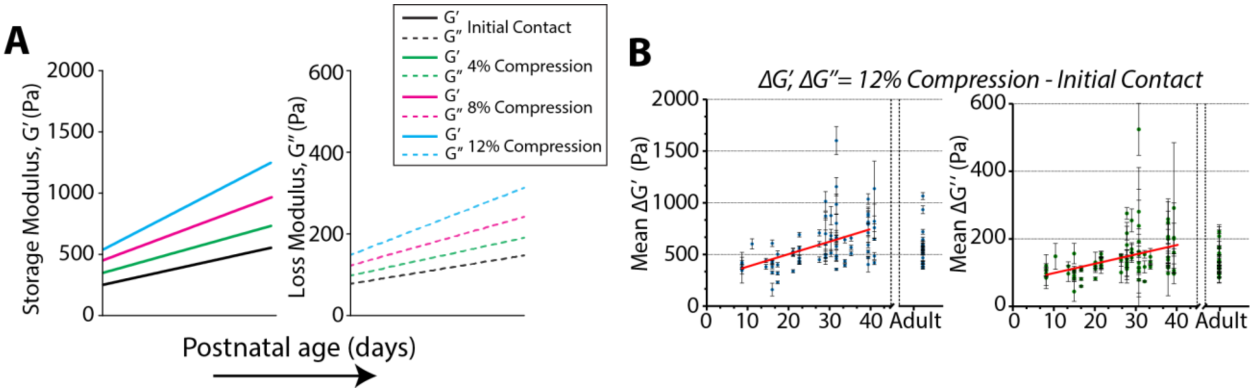

Supplmentary Figure S2. Elastic and viscous components of bulk brain stiffness show differential rates of increase with compression and age. (A) Linear fit lines for G' and G'' replotted from Figure 3. **(B)** Change in G' and G'' due to compression of rheometer. Differences ($\Delta G'$ and $\Delta G''$) are between 12% compression and initial sample contact (greatest and least). Red lines represent linear fit. N $\geq$ 3.

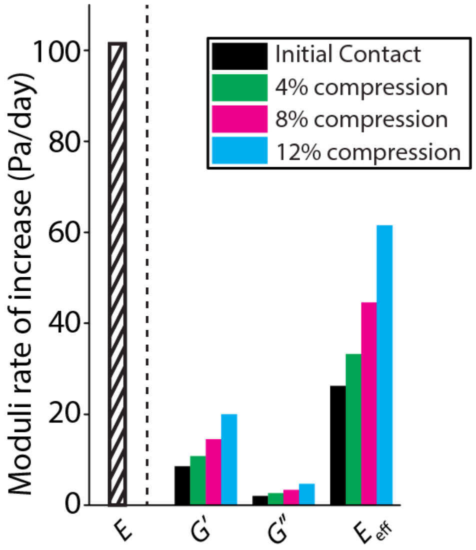

Supplementary Figure S3. Rate of stiffness development in brain tissue differs on global and micro-scales. Comparison of rates of increase in moduli (Pa/day) among AFM (E), Storage modulus (G'), Loss modulus (G''), and effective Young's modulus (E_{eff}, calculated from rheometric data).
